# Incidence of Post-Sedation Emesis in Cynomolgus (*Macaca fascicularis*) and Rhesus (*Macaca mulatta*) Macaques, and Evaluation of Prophylactic Antiemetic Efficacy

**DOI:** 10.3390/ani15223292

**Published:** 2025-11-14

**Authors:** Rachel Coley, Sierra D. Palmer, Jennifer Hubbard, Melanie L. Graham

**Affiliations:** 1 Research Animal Resources, University of Minnesota, Minneapolis, MN 55455, USA; 2 Charles River Laboratories, Cambridge, MA 02142, USA; 3 Department of Surgery, University of Minnesota, Minneapolis, MN 55455, USA

**Keywords:** rhesus macaque, cynomolgus macaque, emesis, sedation, maropitant citrate, ondansetron, antiemetic

## Abstract

Emesis is one of the most common side effects of ketamine sedation; however, predictors like species and sex have not been studied in macaques. This study aimed to determine the incidence and predictors of emesis in macaques undergoing ketamine sedation and to assess the efficacy of orally administered antiemetics in preventing emesis. A retrospective analysis was performed using medical records from rhesus and cynomolgus macaques who received ketamine sedation for veterinary examination events from August 2023 to April 2024. Among the 70 sedations performed using IM ketamine, the overall incidence of emesis was 26%. Cynomolgus macaques had a significantly higher risk of emesis (odds ratio (OR) 46.14, 95% confidence interval (CI): 7.08–493.9; *p* < 0.0001), with an emesis incidence of 55%. Females showed a higher incidence of emesis (75%) with an odds ratio of 3.72 (95% CI: 0.55–17.91; *p* = 0.24), though this difference was not statistically significant. In a prospective, randomized study, we compared the prophylactic use of oral maropitant citrate at a dose of 2 mg/kg or oral ondansetron at a dose of 1 mg/kg versus placebo for the prevention of emesis in cynomolgus macaques undergoing veterinary examination. Emesis was reduced from 58% in the control group to 50% in the maropitant group and 33% in the ondansetron group. However, the reduction in relative risk with either treatment did not reach statistical significance. Prophylactic oral administration of maropitant appears ineffective in preventing emesis in cynomolgus macaques. In contrast, prophylactic oral ondansetron is a more effective alternative in preventing emesis in these animals.

## Simple Summary

A retrospective analysis of 70 ketamine sedation events in rhesus and cynomolgus macaques identified this species as a predictor of emesis, with cynomolgus macaques having a higher incidence of emesis (55%) than rhesus macaques (2.6%). Female cynomolgus macaques had a higher incidence of emesis than males. Next, we conducted a prospective study to assess the efficacy of antiemetics in preventing vomiting. Maropitant citrate, ondansetron, or a placebo was given to cynomolgus macaques prophylactically before a sedation event. Emesis was reduced from 58% in the control group to 50% in the maropitant group and 33% in the ondansetron group. Although the reduction in relative risk was not statistically significant, orally administered ondansetron demonstrated a clinically meaningful reduction in the incidence of vomiting in cynomolgus macaques following ketamine sedation.

## 1. Introduction

Chemical restraint, often using ketamine as the sole agent to induce sedation, is commonly employed for medical and research-related procedures such as veterinary physical exams, disease screening (e.g., tuberculosis (TB) screening), imaging, or biological sample collection in nonhuman primates (NHPs). Training animals to voluntarily participate in medical procedures is an effective strategy to minimize the need for sedation [1]. However, sedation remains essential for procedures that may cause discomfort or require immobility to ensure the safety of both the animal and personnel in certain situations. Side effects of ketamine administration, such as nausea and vomiting, are common in humans [2,3], and vomiting and decreased food intake have been documented in NHPs [4,5]. These side effects may impact the overall comfort of the animal, as well as increase the risk of more severe complications, such as aspiration pneumonia [6,7]. In the context of biomedical research, such occurrences can interfere with data collection by introducing variability. In pediatric medicine, the use of an antiemetic for ketamine sedation is widely recommended [8,9,10]. Reducing emesis post-sedation in NHPs through the use of antiemetics, therefore, provides an opportunity for refinement in animal care and welfare, bringing veterinary standards closer to those applied in human medicine and increasing the translatability of NHP research.

Rhesus macaques (*Macaca mulatta*) and cynomolgus macaques (*Macaca fascicularis*) are two of the most commonly used NHP species in research [11]. Despite differences in their biology [12,13], the incidence of emesis following sedation has not been directly compared between these species. However, one study has shown a higher likelihood of emesis in cynomolgus macaques compared to rhesus macaques after neurosurgical procedures [14]. Another previous study reported the incidence of ketamine-induced emesis in cynomolgus macaques to be 7.3–24.4%, with males experiencing a higher incidence of vomiting than females, though statistical significance was not assessed [4]. This underscores the clinical relevance of emesis in cynomolgus macaques, yet no studies have directly compared the efficacy of antiemetic agents in managing this issue around sedation.

The use of antiemetic agents around sedation events, particularly maropitant citrate and ondansetron, is commonplace in companion animal clinical practice. Maropitant citrate is often chosen due to its efficacy [15,16,17,18,19] and long duration of action [18,20], as well as the availability of oral and injectable veterinary labeled formulations. As a veterinary-specific product not labeled for humans, its use in a biomedical research setting may impact the translatability of the research results. Ondansetron, which has a shorter duration of action [21], is used off-label in veterinary species and is generally considered less effective than maropitant at reducing emesis in dogs, although it may have benefits in controlling nausea [15,21,22,23,24]. However, these findings may not reflect the effectiveness of these agents in NHPs, which are more biologically similar to humans compared to cats and dogs, necessitating further studies to assess potential benefits.

Maropitant is a neurokinin (NK-1) receptor antagonist that works as an antiemetic by inhibiting the binding of substance P in the central nervous system [25]. NK-1 receptors can be found in a range of tissues in both the central nervous system, including in the region associated with the vomiting reflex, and peripheral tissues, including the gastrointestinal tract [26,27]. Because of this wide distribution, NK-1 receptors and substance P have been implicated in a range of pathways such as emesis, pain, inflammation, depression, and anxiety [28]. Maropitant has proven highly effective at reducing vomiting in dogs and cats [15,16,17,18,19]. Although there are case reports describing its perioperative use in macaques [29,30], only one study evaluates its clinical efficacy in this species following neurosurgical procedures [14]. Surgical manipulation introduces variables such as invasive tissue disruption, surgical stress, and the side effects of general anesthesia, all of which may lead to emesis through a variety of mechanisms. The drugs used for sedation, premedication, or in the perioperative period may also contribute to emesis. Drugs like ketamine, an N-methyl-D-aspartate (NMDA) receptor antagonist, may influence vomiting pathways by blocking NMDA receptors in key brain regions involved in nausea, including the vestibular nuclei and the nucleus tractus solitarius. These regions process balance, motion-related nausea, and brainstem vomiting reflexes, suggesting that ketamine may influence emesis through central mechanisms [31,32,33]. Other drugs used for sedation and premedication have also been associated with emesis such as alpha-2-agonists in dogs and cats [34,35,36,37], and opioids in humans, dogs, and cats [37,38,39,40]. Because there are a variety of mechanisms that may contribute to emesis in differing clinical scenarios, antiemetic efficacy must be assessed in each specific context, as treatments that work for one may not be effective for the other.

Ondansetron is a selective 5-HT3 receptor antagonist. While the mechanism of action is not fully understood, it is thought to work as an antiemetic agent by antagonizing serotonin receptors primarily in vagal afferent nerve fibers of the peripheral nervous system and the vomiting center and chemoreceptor trigger zone within the central nervous system [41]. Ondansetron is a widely used antiemetic in human medicine and is effective at managing emesis caused by various conditions, including pregnancy and chemotherapy [42,43,44,45].

This study first evaluated retrospective data to determine whether emesis occurred as a sedation-associated complication in our colony of rhesus and cynomolgus macaques and to assess potential differences in incidence by species or sex. Based on these findings, we prospectively evaluated the effectiveness of prophylactic oral administration of maropitant and ondansetron in preventing emesis in cynomolgus macaques following ketamine sedation. We hypothesized that both antiemetics would reduce the incidence of post-sedation emesis compared to untreated animals, with maropitant expected to be more effective than ondansetron. This expectation was based on maropitant’s broader mechanism of action via NK-1 receptor antagonism, its greater efficacy in dogs compared to ondansetron [15,17], and its regulatory approval and widespread clinical use in veterinary medicine for the prevention and treatment of vomiting, including drug-induced and perioperative emesis [15,16,17,18,19,20,21]. Ondansetron, a serotonin (5-HT3) receptor antagonist widely used in human clinical care, was included to reflect human-relevant treatment protocols. When efficacy is comparable, such agents are often preferred in translational NHP studies to strengthen alignment with clinical practice. Comparing the incidence of emesis between the two treatments to determine their relative safety and efficacy provides valuable insights for improving the welfare of NHPs in captive settings.

## 2. Materials and Methods

### 2.1. Animal Subjects

All animal procedures were approved by the University of Minnesota Institutional Animal Care and Use Committee and adhered to principles stated in the Guide for the Care and Use of Laboratory Animals [46]. Animals were housed and cared for in accordance with the standards set forth in the Animal Welfare Act and Regulations [47,48].

Purpose-bred rhesus and cynomolgus macaques were acquired through institutionally approved commercial vendors. This study was conducted during the qualification phase for study placement, as it involved non-invasive assessments and evaluated procedures consistent with routine veterinary care. Conducting the study at this stage allowed for integration with standard animal handling and monitoring practices, without introducing additional burden or deviation from typical welfare protocols. Animals were housed in same-sex pairs, except during periods of demonstrated social incompatibility, and were kept in facilities fully accredited by AAALAC International. Animals were observed at least twice daily for general appearance and behavior as part of routine health monitoring. Water was provided ad libitum, and biscuits (2055c or 7195 Envigo Harlan Teklad Nonhuman Primate Diet, or 5048 LabDiet Certified Primate Diet) were provided twice daily based on body weight and supplemented with fresh fruits, vegetables, grains, beans, and nuts for enrichment. Room temperature was maintained at 20–26.7 degrees C, humidity was maintained at 30–70%, and lights were programmed to a 12 h-on, 12 h-off circadian light cycle with 30 min dawn/dusk intervals. Weights were collected at least once a month, veterinary rounds were performed weekly for routine evaluation, and physical exams performed by a veterinarian, with bloodwork including complete blood count and serum chemistry, were performed at least annually.

### 2.2. Retrospective Observational Study

**Figure 1:**
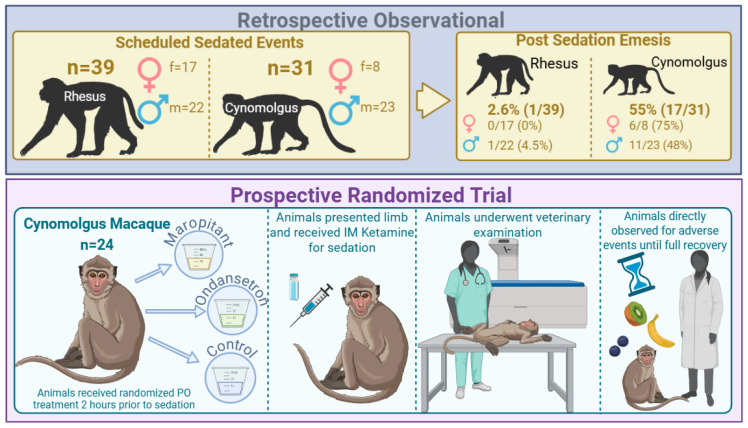
Study design, including a retrospective observational study and a prospective randomized trial. Animals in the retrospective observational study were administered ketamine for a scheduled sedated event, and emesis events were recorded post-sedation. In the prospective trial, animals were given randomized oral treatments (maropitant, ondansetron, or control) 2 h prior to sedation, shown as medication cups to indicate treatment groups and oral administration. The animals received ketamine and underwent veterinary examinations. The hourglass represents the recovery period during which emesis was monitored, and animals could consume food, indicated by food icons.

A retrospective observational analysis was conducted on rhesus and cynomolgus macaques who underwent non-invasive procedural sedation for veterinary exam and/or TB testing within our facility between 30 August 2023 and 17 April 2024 (Figure 1). All animals that underwent sedation with IM ketamine were included. Clinical and sedation records were reviewed to collate relevant data. All animals were fasted for approximately 2 h prior to sedation events, but access to water was continuously provided. Demographic information, including species, sex, age, and weight, was examined together with the ketamine dose and the occurrence of vomiting. Data were analyzed for the incidence of emesis and to determine the demographic predictors of emesis.

### 2.3. Prospective Randomized Trial

Based on retrospective analysis, emesis was not a significant concern in rhesus macaques; therefore, the prospective trial focused exclusively on cynomolgus macaques. A pre-study power analysis revealed that a minimum of 12 animals per group would be required to detect a 50% reduction in vomiting, using the retrospective incidence of 55% (α = 0.05, and β = 0.2). This anticipated reduction was based on veterinary and human clinical trials, comparing vomiting among treated individuals to controls [8,15]. Using G*Power 3.1.9.6 for MacOS, Fisher’s Exact Test was shown to have 83.11% power to detect this difference. The targeted sample size was fully met.

All male and female cynomolgus macaques scheduled to undergo non-invasive procedural sedation for a veterinary exam, TB testing, and/or a Dual-Energy X-ray Absorptiometry (DEXA) scan were eligible for enrollment if they had a history of adequate sedation with ketamine as the sole agent, a record of accepting oral medication, and no systemic health concerns based on their clinical history and most recent physical examination.

Twenty-four demographically matched animals (16 males, 8 females) were prospectively randomized using block randomization (*n* = 12) with sex-balanced allocation (8 males, 4 females per group) to receive either maropitant or ondansetron (Figure 1). Male weights ranged from 3.93 to 10.13 kg, and female weights ranged from 3.7 to 6.9 kg, with an age range of 4.4–10.3 years old. Twelve animals from either group were further randomized to serve as controls, allowing for comparison of their responses to the antiemetics versus no treatment. Animals enrolled in both the treatment group and the control group had a minimum of two weeks between sedation events.

All food was removed approximately 2 h prior to sedation events. Water was available at all times. At least two hours and not more than four hours before routine sedation events, animals were administered either 2 mg/kg maropitant (as citrate) oral suspension (Wedgewood Pharmacy, Swedesboro, NJ, USA), 1 mg/kg ondansetron (as HCl anhydrous oral solution) (Wedgewood Pharmacy, Swedesboro, NJ, USA), or a placebo (flat 1.5 mL of plain water) orally in a small piece of crustless white bread. Drug doses were based on a contemporaneous weight. Animals presented their hind limb on cue from their home enclosure, which allowed for intramuscular administration of 10 mg/kg ketamine for sedation; the time of ketamine administration was recorded. Once appropriately sedated, all animals received abdominal and urogenital palpation by the same veterinarian, blinded to the treatment group, to ensure no confounding variable related to physical manipulation. Animals were positioned in dorsal, left lateral, and right lateral recumbency as required to complete the physical examination. During recovery, animals were initially placed in a forward-facing, upright position to minimize the risk of vomiting and were able to move freely and change position as they regained mobility. Heart rate, respiratory rate, body temperature, vomiting events, and recovery time were recorded for each animal. Animals were monitored continuously until fully recovered, with observers recording all observations on a blinded form. Vomiting events were defined as the active process of retching accompanied by the production of vomitus. The end of a vomiting event was defined as a clear cessation of retching and vomitus production; if vomiting subsequently resumed, it was counted as a new event. A limitation of this definition is that vomiting events in which the animal immediately swallowed the vomitus may have gone unobserved. Criteria for full recovery and discharge from direct observation required animals to be alert and responsive to stimuli, show no signs of pain or distress, have no vomiting for at least 15 min, and exhibit a good appetite and coordinated fine motor control. The time of ketamine administration and recovery time were used to calculate the total time to recovery (in minutes). After discharge, animals returned to the regular observation schedule.

### 2.4. Statistical Analysis

For the retrospective study, risk factors included in the univariate analysis for predicting vomiting post-sedation were species and sex. Additionally, multivariable logistic regression was used to analyze various factors such as age, weight, and ketamine dose on post-sedation vomiting. Animals that experienced a vomiting event were compared with those that did not, and an odds ratio was calculated using Fisher’s Exact Test. For the prospective study, animals were treated with either maropitant or ondansetron. Those who experienced a vomiting event were compared with those who did not, and the relative risk (RR) was calculated using Fisher’s Exact Test. A one-way ANOVA was performed to determine differences in heart rate, respiratory rate, body temperature, total number of vomiting events, and recovery time between each of the three groups. Results were considered significant if *p* < 0.05 (*). All analyses were performed using GraphPad Prism version 10.4.0 (Boston, MA, USA).

## 3. Results

### 3.1. Retrospective Observational Study

A total of 39 rhesus macaque (male = 22, female = 17; 3.1–10.2 years old; 3.68 kg–9.79 kg) and 31 cynomolgus macaque (male = 23, female = 8; 3.7–9.5 years old; 2.95 kg–10.13 kg) routine sedation event records were collected to determine the incidence of sedation-induced emesis by species and by sex within the different species. Ketamine was the sole agent used in each event, and the dose ranged from 10 to 12 mg/kg for rhesus and 6–10 mg/kg for cynomolgus macaques. Due to the limited number of post-sedation vomiting events in rhesus macaques, logistic regression to assess the impact of age, weight, and ketamine dose was not possible. In male cynomolgus macaque, ketamine dose (10 mg/kg odds ratio (OR): 0.11, 95% confidence interval (CI): (0.0009–5.7), *p* = 0.27; 8 mg/kg OR: 0.16, 95% CI: (0.002–5.9), *p* = 0.34) and weight (OR: 41.7, 95% CI: (3.3–11,299), *p* = 0.054) did not influence post-sedation vomiting. We were unable to perform logistic regression, including the 6 mg/kg ketamine dose, due to the sample size being too small. Male cynomolgus macaques that were older in age were less likely to vomit post-sedation (OR: 2.06, 95% CI: (0.0002–0.31), *p* = 0.04). In female cynomolgus macaques, age (OR: 0.17, 95% CI: (3.77 × 10^−5^–47.6), *p* = 0.63) and weight (OR: 0.74, 95% CI: (2.377 × 10^−6^–9.0), *p* = 0.46) did not influence post-sedation vomiting. We were unable to perform a logistic regression model that included the ketamine dose because almost all animals received the same 10 mg/kg dose.

Among the 70 sedations performed, the overall incidence of emesis was 26% (Table 1). The incidence of post-sedation vomiting in rhesus macaques was 2.6% (*n* = 1). Cynomolgus macaques had a significantly higher risk of emesis (OR: 46.14, 95% CI: (7.08–493.9); *p* < 0.0001), with an incidence of emesis of 55% (*n* = 17). Female cynomolgus macaques had a higher observed incidence of emesis (75%), with an odds ratio of 3.72 (95% CI: (0.55–17.91); *p* = 0.24), though this difference did not reach statistical significance and may reflect the limited sample size.

### 3.2. Prospective Randomized Trial

**Figure 2:**
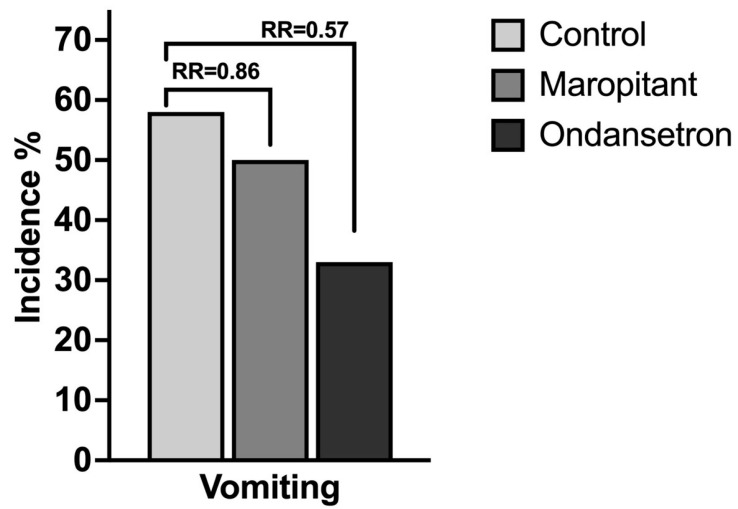
Incidence of emesis for maropitant and ondansetron versus placebo in cynomolgus macaques. Incidences of vomiting are presented (%), along with relative risk (RR). Both maropitant and ondansetron reduced the risk of vomiting, with an RR of 0.86 (0.3924 to 1.819) and 0.57 (0.2173 to 1.377), but this reduction was not significant.

All 24 animals completed the study without adverse events outside of expected emesis, allowing for full evaluation of emesis incidence following administration of maropitant or ondansetron. An appropriate plane of sedation was achieved in all animals with the initial dose of ketamine, and no animals were given additional doses. Following ketamine sedation, vomiting occurred in 58.3% (*n* = 7) of cynomolgus macaques receiving a placebo compared to 50.0% (*n* = 6) receiving maropitant and 33.3% (*n* = 4) receiving ondansetron. Pairwise comparisons using Fisher’s Exact Test revealed no statistically significant differences in vomiting incidence between animals receiving maropitant and those receiving placebo (*p* = 1.000), or ondansetron and placebo (*p* = 0.414). There was also no significant difference between the maropitant and ondansetron treatment groups (*p* = 0.680). We also calculated the relative risks to contextualize the magnitude of effect (Figure 2). When vomiting occurred, the number of vomiting events per animal did not differ significantly between groups (placebo = 2.0 ± 1.29 StD, maropitant = 1.5 ± 0.55 StD, ondansetron = 2.0 ± 0.82 StD; *p* = 0.6151).

Results of the ANOVA are shown in Table 2. Body temperature was lower in the placebo controls than in animals treated with an antiemetic; a Tukey multiple comparisons test showed a significant difference in body temperature between the maropitant and the control groups (*p* = 0.0010), and between the ondansetron and the control groups (*p* = 0.0147), but no significant difference between the two treatment groups (*p* = 0.5672). Although not statistically significant (*p* = 0.4252), the ondansetron group recovered nearly an hour faster than the maropitant group, a clinically meaningful difference given the importance of minimizing recovery time.

## 4. Discussion

In this study, cynomolgus macaques demonstrated a higher incidence of emesis following sedation compared to rhesus macaques, in which vomiting was rare. Although not statistically significant, the higher incidence of emesis observed in female cynomolgus macaques may suggest a possible sex-related susceptibility, though this finding should be interpreted cautiously given the limited sample size. While the species-based differences in emesis seen here are supported in the literature [14], the potential increased risk experienced by female cynomolgus macaques has not been previously observed [4]. A previous study assessing emesis in ketamine-sedated cynomolgus macaques observed a higher incidence of emesis in male cynomolgus macaques compared to females; however, no statistics were run to determine significance. The 1982 study reported an overall emesis incidence of 22.0% in macaques given 10–14.9 mg/kg ketamine IM, with rates of 30% in males and 14.3% in females [4]. Compared to our study, which observed a higher overall incidence and different sex distribution, these variations may reflect differences in animal demographics, geographic origin (not reported in the earlier study), husbandry conditions, or pre-sedation fasting protocols. Notably, the animals in the 1982 study were fasted for more than 24 h, a duration much longer than in our study, which may have influenced susceptibility to emesis. In humans, differences in gastrointestinal motility have been reported, with females having slower gastric emptying compared to males [49,50,51]. A shortened fasting period may therefore disproportionally impact females if gastric contents are not fully emptied prior to sedation. Given the elevated risk of emesis in cynomolgus macaques following ketamine sedation, this study aimed to refine supportive care practices by evaluating the efficacy of orally administered antiemetics, offering a non-invasive alternative to injectable options.

A significant difference in rectal body temperature was observed between the treatment and control groups. Although the cause is unclear, prior studies in humans, rats, and musk shrews have linked motion sickness-induced nausea to mild reductions in body temperature [52,53]. In rats, this effect was prevented by ondansetron administration, suggesting a role for antiemetics in modulating nausea-related hypothermia [53]. While not studied in NHPs, a similar mechanism may be present. In our study, the control group had the lowest body temperatures, possibly reflecting greater nausea compared to the treatment groups. While subcutaneous administration of maropitant at 1 mg/kg has been previously reported in macaques [14,29,30], oral administration has not been documented in this species. In dogs, the standard oral dose is 2 mg/kg and may be increased up to 8 mg/kg to control motion sickness [54]. Given the expected reduction in bioavailability via enteral administration and dosage used in other species, we selected an oral dose of 2 mg/kg for this study.

Oral (PO) administration of ondansetron has been described in macaques, most notably with a standard dose of 4 mg per animal to control nausea associated with diabetes induction [55]. When adjusted for body weight, this corresponds to approximately 1 mg/kg for an average female and 0.5 mg/kg for an average male cynomolgus macaque in our colony. Intravenous and intramuscular routes of administration of ondansetron are more commonly reported than PO in macaques, with doses ranging from 0.1 mg/kg to 2 mg/kg [56,57,58,59]. In dogs and cats, the oral dose can be as high as 1 mg/kg, though this use is considered extra-label in both species [24]. Based on interspecies comparisons and prior institutional experience, we selected an oral dose of 1 mg/kg for ondansetron.

Maropitant is a commonly used veterinary medicine and is FDA-approved for use in dogs and cats [60]. A generic formulation was approved in 2023, improving accessibility to this already popular drug [61]. However, maropitant is much more expensive than ondansetron. As of this writing, a single 16 mg maropitant tablet costs $4.00 compared to $0.20 for a single 4 mg tablet of ondansetron, from a well-known pet pharmacy [62,63]. On a per milligram basis, maropitant is roughly five times more expensive. Even when accounting for ondansetron’s lower dose and more frequent dosing (three times daily vs. once daily for maropitant), ondansetron remains over three times less costly than maropitant.

Additionally, maropitant is labeled only for veterinary use. Another NK-1 receptor antagonist, aprepitant, is approved for human use, but it is primarily used to treat chemotherapy-induced nausea [64]. In contrast, ondansetron has broader clinical indications [42,43,44,45], making it potentially more suitable for translational research applications.

Although neither anti-emetic treatment reached statistical significance in reducing vomiting, both exhibited a lower incidence of emesis compared to controls. More than half of control animals experienced vomiting after sedation, while only half of maropitant-treated animals and one-third of ondansetron-treated animals did. The study was powered to detect a 50% reduction in emesis, based on prior data in dogs and humans [8,15]; however, the observed effect size was smaller, suggesting a larger sample size would be required to confirm statistically significant differences. In companion animal studies, antiemetic efficacy is often evaluated against pharmacologically induced emesis or within multimodal premedication protocols, which limits direct comparison with the present findings. Nonetheless, the reduction seen in the present study—particularly in the ondansetron group—represents a meaningful improvement that may be beneficial in practice. While additional studies are needed to support widespread adoption, the findings suggest that prophylactic ondansetron may be a useful consideration for institutions aiming to reduce post-sedation emesis in cynomolgus macaques.

The longer recovery time observed after maropitant administration, compared to both the control and ondansetron groups, suggests a need for further studies to explore potential interactions between maropitant administration and ketamine dosing, since maropitant is known to lower the minimum alveolar concentration (MAC) of inhaled anesthetics in dogs and cats [65,66].

The present study examined species and sex as risk factors for emesis and evaluated therapeutic interventions aimed at reducing its incidence. Additional factors influencing post-sedation emesis warrant further investigation to guide non-pharmaceutical approaches to prevention. Fasting duration, for example, may have contributed to the difference in emesis incidence observed between this study and a previous report [4]; accordingly, future research should evaluate fasting duration, including potential sex-related effects, as a risk factor for post-sedation emesis. Furthermore, older male cynomolgus macaques were less likely to vomit than younger males. As weight did not influence emesis risk, this finding may reflect greater prior exposure to ketamine and possible tolerance development. While this relationship was not assessed here, future studies should examine whether repeated ketamine administration affects emesis susceptibility.

Because the reduction in emesis observed in this study was smaller than that reported in prior human and animal studies [8,15], future work could explore whether alternative routes of administration, such as intravenous, intramuscular, or subcutaneous, offer greater efficacy. Oral administration was selected to minimize injection-related discomfort and handling stress; if efficacy is comparable across routes, the oral route would remain preferable in accordance with refinement principles. Finally, as the doses and timing used here were based on limited macaque-specific data and largely extrapolated from other species, further optimization may help reduce emesis. Administering an injectable antiemetic after sedation has been achieved, for instance, could minimize both vomiting and the distress associated with pre-sedation injections.

## 5. Conclusions

This study highlights an opportunity to refine sedation protocols by recognizing species- and sex-specific differences in emesis risk, particularly in cynomolgus macaques. Rather than relying on a one-size-fits-all approach, these findings support evidence-based, context-specific strategies for the use of antiemetics. Evaluating macaques for their risk of emesis during sedation in captive settings enables the targeted application of antiemetics, reducing complications and improving animal welfare. This approach not only enhances procedural consistency and reduces variability in data collection but also contributes to the broader goals of refinement. Ultimately, it strengthens both animal welfare and the scientific integrity of studies involving NHPs.

## Figures and Tables

**Table 1:** Incidence of post-sedation emesis by species and sex. Values are presented as number/total (percentage).

	All Animals	Cynomolgus	Rhesus
Total	18/70 (26%)	17/31 (55%)	1/39 (2.6%)
Male	12/45 (27%)	11/23 (48%)	1/22 (4.5%)
Female	6/25 (24%)	6/8 (75%)	0/17 (0%)

**Table 2:** Vital signs and recovery time following ketamine sedation, with or without an anti-emetic. Results presented as mean ± standard deviation. Groups labeled with letters a and b differed statistically. NS indicates not statistically significant (*p* ≥ 0.05).

	Control (*n* = 12)	Maropitant (*n* = 12)	Ondansetron (*n* = 12)	*p*-Value
Heart Rate (beats per minute)	137.3 ± 18.0	135.2 ± 24.41	143.0 ± 26.26	NS
Respiratory Rate (breaths per minute)	25.33 ± 5.21	25.67 ± 3.6	25.00 ± 4.22	NS
Body Temperature (°F)	98.94 ± 0.84 ^a^	100.3 ± 0.63 ^b^	99.96 ± 1.0 ^b^	0.0010
Time to Recovery (minutes)	198.7 ± 109.5	213.9 ± 106.2	162.0 ± 77.11	NS

## Data Availability

The raw data supporting the conclusions of this article will be made available by the authors on request.

## Abbreviations

The following abbreviations are used in this manuscript:

NHP	Nonhuman Primate
TB	Tuberculosis
DEXA	Dual-Energy X-ray Absorptiometry
MAC	Minimum alveolar concentration
PO	Per os
